# Acute Exacerbation of Chronic Obstructive Pulmonary Disease: Cardiovascular Links

**DOI:** 10.1155/2014/528789

**Published:** 2014-03-02

**Authors:** Cheryl R. Laratta, Stephan van Eeden

**Affiliations:** ^1^Department of Medicine, University of Alberta, Edmonton, AB, Canada; ^2^UBC James Hogg Research Center, Institute for Heart and Lung Health, University of British Columbia, Canada; ^3^Respiratory Division, Department of Medicine, St. Paul's Hospital, Vancouver, BC, Canada

## Abstract

Chronic obstructive pulmonary disease (COPD) is a chronic, progressive lung disease resulting from exposure to cigarette smoke, noxious gases, particulate matter, and air pollutants. COPD is exacerbated by acute inflammatory insults such as lung infections (viral and bacterial) and air pollutants which further accelerate the steady decline in lung function. The chronic inflammatory process in the lung contributes to the extrapulmonary manifestations of COPD which are predominantly cardiovascular in nature. Here we review the significant burden of cardiovascular disease in COPD and discuss the clinical and pathological links between acute exacerbations of COPD and cardiovascular disease.

## 1. Introduction

Chronic obstructive pulmonary disease (COPD) is characterized by emphysema, small airways disease, and bronchitis, associated with pulmonary hypertension. The chronicity of COPD is well documented, characterized by a progressive decline in lung function associated with airway narrowing due to inflammation, fibrosis and mucus plugging, and parenchymal destruction with a loss of elasticity, gas exchange surface area, and airway support with subsequent early airway closure [1]. Acute insults result in the clinical syndrome of acute exacerbation of COPD (AECOPD) where the classical etiology is either infectious, viral or bacterial, or environmental in nature [1]. Not infrequently, there is a cardiovascular trigger underlying the clinical presentation, and this remains challenging to identify [2]. It is imperative that we further explore our understanding of AECOPD as this clinical diagnosis constitutes a major cause of morbidity and mortality in the COPD population, with a 50% mortality at 3.6 years, a 75% mortality at 7.7 years, and a 96% mortality at 17 years following the index hospitalization for AECOPD [3]. In this review, we will explore what we understand about the relationship between cardiovascular disease and AECOPD. We will also explore how current treatments for AECOPD impact cardiac disease and vice versa in order to improve management for patients with AECOPD.

## 2. Cardiovascular Disease and COPD: Friend or Foe?

### 2.1. Epidemiology

The close association between COPD and cardiovascular disease has received significant attention in the last fifteen years in a concerted effort to improve our understanding of the systemic consequences of COPD. It is estimated that the diagnosis of COPD increases the risk of cardiovascular disease by an OR of 2.7 (95% CI 2.3–3.2) [4]. Finkelstein and colleagues report that patients with COPD are at a significantly higher risk of coronary artery disease (OR 2.0, 95% CI 1.5–2.5), angina (OR 2.1, 95% CI 1.6–2.7), myocardial infarction (OR 2.2, 95% CI 1.7–2.8), stroke (OR 1.5, 95% CI 1.1–2.1), and congestive heart failure (OR 3.9, 95% CI 2.8–5.5) [4]. Not unexpectedly, those hospitalized for AECOPD have a high prevalence of coexisting cardiovascular disease, often exceeding 50% [5]. These associations have been documented in a variety of different nationalities and ethnicities, including those within North America [4, 6–13], Asia [5], South America [14], and Europe [15–19], to list a few.

Mortality from cardiovascular disease is similarly increased in the COPD population. The 45,966 patients with COPD in the Northern California Kaiser Permanente Medical Care Program had an adjusted RR for mortality for all cardiovascular endpoints of 1.68 (95% CI 1.50–1.88), ranging from 1.25 (stroke) to 3.53 (heart failure) [6]. Both the Buffalo Health Study and the Lung Health Study, two well-described prospective studies, found an increased mortality from ischemic heart disease associated with degree of airway obstruction on spirometry [7, 8]. A pooled estimate of large population studies published before 2005 estimates that the RR of cardiovascular mortality is 1.99, 95% CI 1.71–2.29 [9].

COPD patients do not tolerate cardiac injury or intervention as well as those without airways obstruction. Bursi et al. determined that COPD subjects with an acute myocardial infarction have a five-year survival rate of 46% (95% CI 41–52%) as compared to those without COPD (survival rate 68%, 95% CI 66–70%), with an adjusted hazard ratio of 1.30, 95% CI 1.10–1.54 [20]. The VALIANT trial had similar results in their population, with an HR for all-cause mortality of 1.14, 95% CI 1.02–1.28 [21]. Salisbury et al. looked at subjects with obstructive airways disease following index myocardial infarction and document an elevated one-year mortality (HR 2.00; 95% CI 1.44–2.79) and a lower-health related quality of life, as compared to those without obstructive lung disease [11]. Coexisting COPD confers an increased risk for all-cause mortality when undergoing coronary artery bypass grafting as documented by Angouras et al. and Leavitt et al. (HR 1.28, 95% CI 1.11 to 1.47 and 1.8, 95% CI 1.6–2.1, resp.) [22–24].

The difference in mortality after a cardiovascular event may relate to a difference in management of COPD patients with cardiovascular disease. Those with coexisting cardiac disease and COPD are more likely to have less aggressive treatment with cardiac medications and/or coronary angiography [11, 20, 25, 26]. Furthermore, on discharge, patients with obstructive airways disease are less likely to receive percutaneous coronary intervention (50.9% versus 62.9%), to be discharged on aspirin (87.8% versus 94.5%) or a *β*-blocker (86.2% versus 92.6%), or to be referred for cardiac rehabilitation (37.5% versus 50.4%) following an index myocardial infarction than those without obstructive airways disease [11].

### 2.2. COPD and Classical Cardiovascular Risk Factors

COPD contributes to cardiovascular risk, so it is important to understand whether other cardiovascular risk factors are associated with COPD as well. There are a multitude of studies linking classical cardiovascular risk factors [27, 28] to COPD, such as hypertension, a family history of coronary artery disease, an abnormal lipid battery, or diabetes [6, 10, 20, 29]; yet other studies demonstrate no independent association but simply a high prevalence of these conditions [30]. Two classical risk factors for coronary artery disease, age and male gender, are associated with increased mortality in COPD [3]. The diagnosis of COPD is associated with a higher prevalence of a 10-year cardiovascular risk assessment >20% in those aged 55–74 than the general population [10]. Notably, though, even the young or female patients with COPD experience significant cardiovascular morbidity [6]. At this time, studies strongly support the association of COPD with smoking and age as the most prominent classical cardiovascular risk factors associated with the COPD population [15, 20], and it is recognized that classical cardiovascular risk factors and COPD commonly coexist.

Interestingly, the metabolic syndrome phenotype is commonly present in COPD patients [31, 32], yet a low body mass index (BMI) carries a worse prognosis [33–35]. A low BMI in COPD patients is linked to increased systemic inflammation [36], and it may be systemic inflammation driving cardiovascular disease rather than the negative health effects of obesity. A surrogate biomarker for systemic inflammation, C-reactive protein (CRP), is independently associated with both all-cause and cardiovascular mortality in this population (RR 1.79 95% CI 1.25–2.56, RR 1.69 95% CI 1.86–3.33, resp. when the highest quintile of CRP is compared to the lowest) [37]. Further work is needed to determine the benefit to an elevated BMI in advanced COPD and the implications to the cardiovascular system in early stages of COPD when obesity is commonly present.

### 2.3. Nature of Cardiovascular Disease in Subjects with COPD

#### 2.3.1. Systemic Vascular Disease and COPD

The presence of peripheral vascular disease is very common in COPD patients, and there are multiple variables that contribute to this disease process including the systemic inflammatory response induced by the inhalation of cigarette smoke, diesel exhaust particles, and other air pollutants [38]. Peripheral vascular disease has been documented in the upper extremities [39–43] and the large, central vessels, such as the carotid and femoral arteries [44–47]. Multiple methodologies to document increased arterial stiffness are validated, either relating vessel size adjustments to distending pressure, determining pulse wave velocity (PWV), or examining pulse waveforms [48]. Aortic PWV (utilizing carotid-femoral distance in equation or the carotid-femoral distance after subtraction of sternal-femoral distance or carotid-sternal distance) is strongly associated with cardiovascular events, cardiovascular mortality, and all-cause mortality in the general population [49]. It is also a safe and noninvasive tool that has the advantage of being highly reproducible in the COPD population [45, 50]. The frequency of increased intima-media thickness and abnormal PWV increases as FEV1 decreases [44, 46]. There is mounting evidence that endothelial cell dysfunction, as measured through surrogates such as flow-mediated dilation, correlates with severity of COPD [40–42, 51, 52].

Cerebrovascular disease, as a manifestation of severe vascular disease, is increased in the COPD population, both in the presence of acute inflammation and in chronic disease [53]. A cross-sectional analysis of COPD patients identifies that COPD confers the highest increase in risk of stroke to those in the lowest quintile of age (age 35–44, HR 3.44, 95% CI 0.85–13.84), with the oldest quintile of age having the lowest increase in risk (age ≥ 75; HR 1.10, 95% CI 0.98–1.23) [54]. The burden of strokes is inversely proportional to FEV1 [53], although there have been some studies that do not find this association independent of other risk factors [26].

#### 2.3.2. Pulmonary Vasculature and Right Heart Dysfunction in COPD

The emphysematous component of COPD is characterized by destruction of alveolar walls and pulmonary capillaries, hyperinflation with resultant positive alveolar pressure throughout inspiration, hypoxic vasoconstriction, and pulmonary vascular endothelial dysfunction, with subsequent pulmonary hypertension. Depending on the definition used, 25 to 70% of COPD patients have pulmonary hypertension [1, 55–57]. Pathological changes of pulmonary hypertension are present in tissue samples from COPD patients who do not have a diagnosis of pulmonary hypertension [58, 59]. It is estimated that 25% of patients with moderate to severe COPD will develop pulmonary hypertension within 6 years, if they have no disease at baseline [60]. Severe pulmonary hypertension, defined by a mean pulmonary artery pressure (PAP) of greater than 40 mmHg, accounts for <5% of diagnoses and is usually disproportionate to the degree of airflow obstruction [57]. The majority of these patients will have a comorbid disease that is contributing to the degree of pulmonary hypertension [61]. In the right heart, objective findings that have been detected in COPD patients include concentric right ventricular (RV) hypertrophy and elevated end-diastolic RV pressures, one of the first manifestations of adjustment to elevated pulmonary artery pressures, followed by impaired relaxation and systolic dysfunction [57, 62]. RV systolic dysfunction is common in end-stage COPD, with one study documenting an average RV ejection fraction of 45 ± 9% [63]. Both right heart failure and pulmonary hypertension are associated with increased mortality in COPD patients [64].

#### 2.3.3. Left Heart Dysfunction and COPD

Left heart dysfunction in COPD patients can be a challenge to recognize clinically and has recently been shown to be associated with significant morbidity and increased mortality in the COPD population. Many of the early studies looking at cardiac dysfunction and COPD lacked objective parameters defining reproducible echocardiographic measures and spirometric criteria [65]. In those with peripheral vascular disease, mild COPD is associated with subclinical left ventricular (LV) dysfunction (OR 1.6, 95% CI 1.1–2.3), but only moderate-severe COPD is associated with subclinical LV dysfunction and clinical heart failure (OR 1.7, 95% CI 1.2–2.4, and OR 2.0, 95% CI 1.2–3.6, resp.) [18]. In this population, subclinical LV dysfunction increased all-cause mortality in all patients with COPD, with risk the least in those with mild COPD (HR of 1.7, 95% CI 1.2–5.9) and most in those with moderate to severe COPD and overt heart failure (HR of 3.8, 95% CI 1.6–9.1) [18]. Studies have estimated that in patients with moderate COPD, up to 20.5% have unrecognized heart failure with reduced or preserved ejection fraction by their care providers [14, 17]. Similarly, the diagnosis of airflow obstruction is commonly missed in patients with cardiac disease [14, 66], indicating that recognition of these comorbid diseases requires a high index of suspicion.

COPD patients with co-existing heart failure experience similar mortality, hospitalization for cardiovascular events, and frequency of pulmonary events, regardless of whether they have a preserved or reduced ejection fraction [67]. Interestingly, classical examination features that are associated with an acute exacerbation of heart failure in the general population are valid in the COPD population. Physical examination findings associated with heart failure with comorbid obstructive airways disease include presence of rales, leg edema, elevated jugular venous pressure, and an S3 [68].

#### 2.3.4. Arrhythmias and COPD

Arrhythmias associated with airflow obstruction are relatively common and have been a long-standing research interest. Hudson et al. in 1973 report a variety of arrhythmias noted in those with airflow obstruction, as defined by ATS guidelines in the 1970's [69]. More recently, 35.6% of COPD patients who underwent 24-hour Holter monitoring at age 68 had frequent or complex ventricular arrhythmia, as defined by Lown classes 2–5, recorded at baseline [70]. Frequent or complex ventricular arrhythmia on this recording was significantly associated with increased severity of obstructive airways disease, higher mortality (52% versus 41%), and coronary event rates (28% versus 18%) over 14 years of follow-up [70]. This data supports the known association of FEV1 with risk of cardiovascular death, ischemic heart disease, and mortality but also identifies the correlation of these with increased ventricular ectopy and ventricular arrhythmia.

An increased risk of supraventricular arrhythmia has also been reported, most commonly atrial fibrillation and multifocal atrial tachycardia. A higher risk of irregular heartbeats [4] and postoperative supraventricular tachycardia [71] has been reported. Atrial fibrillation occurs in an estimated 8 to 13% of all admissions to hospital for AECOPD [72, 73], with lower prevalence (~2%) of patients with stable disease followed longitudinally [74, 75]. In those with COPD, mean age in the early 50 s, approximately 0.4% will be found to have atrial fibrillation on re-examination at 5-years with an additional 2.2% identified to have atrial fibrillation in the emergency department within that 5 year period [74]. Presence of atrial fibrillation has been found to be inversely associated with FEV1 [74]. Multifocal atrial tachycardia (MAT) is a less common arrhythmia but highly associated with COPD. The estimated in-hospital prevalence of MAT in the general population is 0.05% to 0.32% with 55–66% of these diagnoses associated with comorbid COPD [76, 77]. The estimated in-hospital mortality with MAT is 45% and up to 80% in those with COPD likely due to the severity of acute illness and significant comorbidity in this population [76]. MAT is underdiagnosed on electrocardiogram (ECG) as it is commonly misinterpreted as atrial fibrillation [78].

## 3. Acute Exacerbations of COPD and Cardiovascular Disease

### 3.1. Cardiovascular Disease and Mortality in AECOPD

While numerous factors have been associated with poor outcomes from AECOPD, cardiovascular disease is being increasingly recognized as an important predictor of in-hospital mortality. Cardiovascular risk factors and cardiac comorbidities that correlate with in-hospital mortality include age [73, 79–82] and male gender [79], cerebrovascular disease [73], ischemic heart disease [83, 84], atrial fibrillation [73], and congestive heart failure [84]. Cardiovascular features on admission that aid prognostication are hypotension, tachycardia, arrhythmia, stroke, pulmonary edema, elevated mean PAP > 18 mmHg, and bilateral pedal edema [81, 84–86]. Cardiac biomarkers associated with in-hospital mortality include an elevated serum CRP [73], serum troponin [83–85], NT-pro-brain natriuretic peptide (NT-proBNP) [85], and brain natriuretic peptide (BNP) [55]. Further studies into prediction models that can be utilized in clinical practice for prognostication are necessary to translate this knowledge into clinical care that will improve patient outcomes.

The cardiovascular morbidity of the population admitted with AECOPD is often underappreciated and underdiagnosed at the time of admission, as it is in stable disease. As an example, a high frequency (55%) of those with AECOPD have systolic or diastolic left ventricular dysfunction; more than are accounted for by known comorbid disease [87]. In the TORCH trial, there was a high frequency of sudden death, but disproportionately low prevalence of documented myocardial infarction, leading them to speculate that this was a diagnosis that may have been missed [88]. This is plausible, particularly since COPD patients have an increased risk of acute myocardial infarction (unadjusted HR 3.53, 95% CI 3.02–4.13) [54] and a high frequency of electrocardiographic evidence of previous myocardial infarction with no known ischemic heart disease [89]. A retrospective review of 43 autopsies of patients who died within 24 hours from admission from AECOPD in a university-affiliated tertiary care facility in Serbia revealed that 37.2% had a final cause of death from heart failure and 20.9% from pulmonary emboli [2]. AECOPD proves itself to be a complex clinical scenario that is difficult to separate from the cardiovascular pathology that is so frequent in this population. Lately, due to this diagnostic challenge, there has been recent discourse on the terminology given to these patients, using “acute respiratory symptoms in patients with COPD” or “exacerbation of respiratory symptoms in patients with multimorbidity,” rather than “acute exacerbation of COPD” [90].

### 3.2. Cardiovascular Disease and Readmission to Hospital following AECOPD

There is much interest in the prevention of repeated or prolonged hospitalizations for AECOPD as recurrent admissions are associated with a higher all-cause mortality [3, 80]. Comorbid cardiovascular disease and an elevated troponin at the time of discharge are independently associated with increased risk of readmission for AECOPD [87]. Cardiovascular modifying factors such as physical activity and a higher physical quality of life score are protective and decrease the risk of readmission [91]. A major predictor of readmission for an AECOPD is the severity of airway obstruction, an independent risk factor for cardiovascular disease [81, 84, 92, 93].

Length of stay in hospital for an acute exacerbation of COPD averages between 6 and 9 days [81, 91, 94]. Predictors of an increased length of stay include age ≥65 years, poor performance status, or lowest FEV1 tertile, on a multivariate analysis of COPD patients [81]. Elevated serum troponin has been associated with longer length of stays for AECOPD (Harvey and Hancox report 5 days, 95% CI 1–20 versus 3 days, 95% CI 1–15) [95, 96].

### 3.3. AECOPD as a Trigger for Cardiovascular Events

There is mounting evidence that associates a higher frequency of acute cardiovascular disease with acute respiratory illness, such as pneumonia or AECOPD. In the general population, subjects with a respiratory tract infection are more likely to get an acute myocardial infarction within 1-2 weeks (OR 2-3) [97–99]. Huiart and colleagues found that current use of oral corticosteroids was associated with an increased risk of an index myocardial infarction (RR 2.01 95% CI 1.13 to 3.58), particularly if the prescription was for 25 mg or greater (RR 3.22, 95% CI 1.42 to 7.34) [100], suggesting that patients were undergoing active treatment for AECOPD at the time of the myocardial infarction. Increased likelihood of cerebrovascular disease is also associated with systemic respiratory tract infection [97, 101]. Some of the literature supports significant reduction in cardiovascular morbidity and mortality with prevention of respiratory tract infections; as an example, Nichol et al. report that influenza vaccinations were associated with a 19% reduction in hospitalization for cardiac disease, 16–23% reduction in cerebrovascular disease, 29–32% reduction in pneumonia or influenza, and 48–50% reduction in all-cause mortality in those ≥65 years of age [102]. Some studies have suggested a decrease in cardiac arrest in the community with vaccination against influenza [103] but a systematic review suggests a nonsignificant protective effect from vaccination against cardiovascular death (RR 0.51, 95% CI 0.15–1.76) [104].

Increased vascular risk associated with infection can be more broadly defined as secondary to an acute inflammatory lung condition, as other inflammatory states within the lung are associated with vascular dysfunction as well. Diesel exhaust has been linked to exercise-induced ST-segment depression and surrogates for vascular endothelial dysfunction [105, 106]. Air pollution has been linked to increased cardiac arrest [107]. Exposure to smoke as a firefighter is associated with significant lung inflammation [108] and an increased frequency of death from coronary artery disease [109]. The association between an acute on chronic inflammatory insult and increased cardiovascular events is explored by Man et al., who describe the possibility that increased systemic inflammation may destabilize vulnerable plaques and induce a prothrombotic state [38]. Such profound endothelial dysfunction occurs during these episodes of acute inflammation that macroscopic surrogates of endothelial and vascular smooth muscle function objectively improve three months following an AECOPD [51], increasing the plausibility of this theory.

## 4. Markers of Cardiac Disease in AECOPD

### 4.1. Electrocardiograms

In any disease state, ECG analyses are used to identify predisposing risk for development of ischemia/arrhythmias or signs of underlying cardiac disease. In stable COPD patients, there are subtle changes in the ECG that are not present in the general population. Screening ECGs in patients with COPD have a low coefficient of variation of the RR interval that correlates with degree of hypoxemia [110]. Depressed heart rate variability, of which the variation of the RR interval is a surrogate, is correlated with mortality after a myocardial infarction in the general population [111]. A series of studies suggest COPD is associated with a disturbance of autonomic function manifested by increased sympathetic activation [112–115] and loss of normal circadian variations in heart rate [116]. Finally, vertical P wave axis correlates with a diagnosis of COPD, as measured by P wave amplitude in lead III > lead I and/or a dominantly negative P wave in lead aVL, findings that indicate a P wave axis >60° [117–119]. As such, vertical P wave axis on ECG has been suggested as a screening tool for unrecognized COPD.

Stable COPD patients (*n* = 243) are more likely to have an abnormal ECG than the general population (OR 1.5; 95% CI 1.0–2.1) as summarized: 11% premature ventricular contractions, 7% atrial fibrillation, 9% prolonged corrected QT (QTc) interval, 7% right bundle branch block (RBBB), 2% left bundle branch block (LBBB), 14% left anterior fascicular block, 0.4% left posterior fascicular block, 10% intraventricular block, 8% atrioventricular block, 1% RAE, 7% left ventricular hypertrophy, 1% right ventricular enlargement (RVH), 10% ST segment depression, 0% T wave abnormalities, and 7% inferior and 4% anterior Q-wave myocardial infarctions [120]. In comparison to normal controls, patients with COPD were significantly more likely to have an LBBB, RBBB, a higher resting heart rate, or a prolonged QTc and less likely to have bradycardia [120]. In those referred for pulmonary rehabilitation, 21% of stable COPD patients had an ischemic ECG at rest [121]. Holtzman and colleagues demonstrated that severe COPD patients are more likely to have RAE, RVH, RBBB, marked clockwise rotation, low voltage in limb leads, inferior QS pattern, left axis deviation, premature atrial contraction, supraventricular tachycardia, and an abnormal ECG as compared to mild/moderate COPD [122].

**Figure 1:**
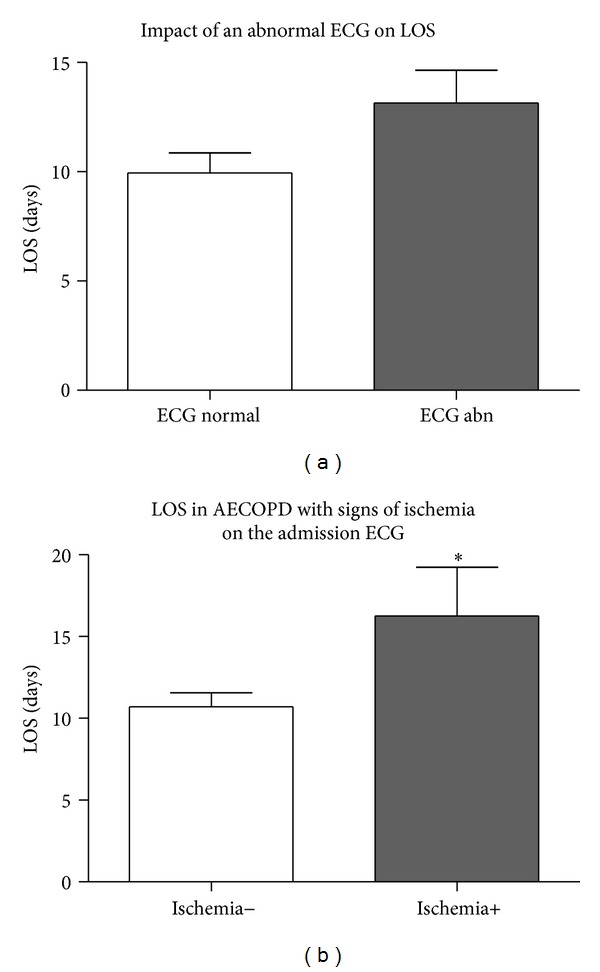
Presence of an electrocardiogram (ECG) abnormality and length of stay (LOS) of subjects admitted with an AECOPD to St. Paul's Hospital or Mount St. Joseph's Hospital between 2007 and 2008. The presence of ECG abnormalities did not influence LOS (11.7 ± 1.4 versus 13.2 ± 1.5, (a) *P* = NS). Subjects with ischemic changes on ECG had a longer LOS (11.2 ± 1.0 versus 16.6 ± 3.0, (b) *P* = 0.031).

In AECOPD, ECG changes are very common, and studies demonstrate a high frequency of finding a new abnormality on ECG from baseline. In a study by Harvey and Hancox, 8% had ST segment depression, 37% had T wave changes, 17% had conduction block, and 6% had a new change on their ECG from baseline (*n* = 182) [96]. ECG findings of a conduction block, ST segment depression, and T wave changes are more likely if the patient has elevated troponin levels [96]. In the emergency department of tertiary care facilities in Vancouver, BC, Canada, we reviewed the ECGs of 163 admissions for AECOPD from 82 patients. Eighty-seven percent of admissions had an ECG, of which 65% were abnormal and 58% had a new abnormality from baseline. ECG abnormalities were as follows: 24% ischemic changes; 17% evidence of a previous myocardial infarction; 8% arrhythmia (excluding tachycardia); 30% heart block; and 16% chamber enlargement (van Eeden, unpublished data). An abnormal ECG, particularly with ischemic changes, on presentation to the emergency department with AECOPD, is correlated with a prolonged hospital stay (see Figure 1, van Eeden, unpublished data). These findings suggest that ischemic ECG changes are associated with more morbidity from AECOPD. There are few studies on electrographic changes in this high risk population, and very little is known about the prognosis and implications of these changes in the acute setting.

AECOPD is associated with greater prolongation of P wave dispersion than in stable COPD [123]. The association of prolongation of P wave dispersion and right atrial enlargement (RAE) with COPD may explain the increased prevalence of atrial arrhythmias, such as atrial fibrillation and MAT [124, 125]. Increased P wave amplitude is present in 14% of patients presenting to the emergency department with AECOPD [126]. P wave amplitude decreases after acute treatment in patients admitted for AECOPD, possibly reflecting reduced right atrial strain [126].

### 4.2. BNP/NT-proBNP

The natriuretic peptides have an established role in differentiating amongst the causes of dyspnea in COPD patients presenting with AECOPD [127]. These biomarkers increase the accurate identification of the trigger of the exacerbation and aid in prognostication while in hospital and after discharge. The BNP Multinational Study identified that in the general adult population presenting to the emergency department with dyspnea, of whom 25% had a history of obstructive airways disease, a BNP of 100 pg/mL had a sensitivity (Sn) of 93.1%, specificity (Sp) of 77.3%, positive predictive value (PPV) of 51.9%, negative predictive value (NPV) of 97.7%, positive likelihood ratio (+LR) of 4.10, and negative likelihood ratio (−LR) of 0.09 [68]. Mueller et al. similarly identified that a BNP of 100 pg/mL had a similar predictive value for the detection of congestive heart failure in patients who present to the emergency department dyspneic with a history of COPD, asthma, pneumonia, or PE [128]. In this study, a BNP of >500 pg/mL was considered to be diagnostic of heart failure, <100 pg/mL excluded heart failure, and 100–500 pg/mL had to be combined with clinical judgement [128]. The use of BNP in the initial assessment resulted in earlier initiation of therapy, reduced hospital admissions, shorter length of stays in hospital, and lower costs of treatment [128]. With use of BNP as outlined, 95% of heart failure subjects are correctly diagnosed rather than 35% [128].

Similarly, NT-proBNP levels in patients with AECOPD and left heart failure were significantly higher than those with AECOPD without LV failure or stable controls [129]. An NT-proBNP of 935 pg/mL has a Sn of 94.4%, Sp of 68.2%, accuracy of 74.3%, and NPV of 97.6%, whereas at a level of 584 pg/mL, heart failure was excluded with a NPV of 100% [129]. Abroug and colleagues identified a similar cut-off of 1000 pg/mL being accurate to rule out left-heart involvement in AECOPD (Sn 94%, NPV 94%, and negative likelihood ratio (−LR) 0.08) [130]. In order to rule in LV involvement in AECOPD, an NT-proBNP cut-off of 2500 pg/mL had the best operating characteristics (+LR 5.16) [130]. In subjects admitted to hospital with an AECOPD, increased NT-proBNP was associated with 30-day mortality (OR 9.0, 95% CI 3.1–26.2) [85] and long-term mortality [87, 131]. Rutten et al. compared BNP and NT-proBNP directly and found that they behaved similarly, able to identify systolic LV dysfunction more frequently than diastolic LV dysfunction or RV dysfunction [132].

The utility of BNP and NT-proBNP is well-proven in its ability to document heart failure, but clinicians are still challenged to predict whether the BNP is indicative of right heart dysfunction or left heart dysfunction. Several studies have attempted to correlate BNP and NT-proBNP with right heart and left heart pathology but have as yet not been able to discriminate between the two [55, 129, 133, 134].

### 4.3. Troponin

Elevated troponin levels are hallmarks of stress or ischemia affecting the myocardium. At baseline, COPD patients have been shown to have higher highly sensitive cTnT than the general population [135]. Patients who present with AECOPD are often found to have elevated serum troponin [136, 137], of which a minority are secondary to an acute coronary syndrome [96, 137–139], often in the absence of chest pain [140]. In those admitted to hospital for an AECOPD, elevated serum troponins are correlated with long-term mortality [83, 95, 138, 139], 30-day mortality [85], in-hospital mortality [142], increased length of stay [95, 96] and risk of readmission [87]. Høiseth et al. found a similar association between mortality and highly sensitive cTnT, most notably in those who were tachycardic (heart rate >100 bpm) [139]. Elevated troponins have been associated with the elderly, comorbid heart failure, chronic renal failure, atrial fibrillation, atrial flutter, increased requirement for NIPPV, and higher BNP levels [95]. Interestingly, elevation in serum troponins in AECOPD has also been associated with presence of neutrophilia, supporting the presence of an inflammatory milieu at the time of troponin elevation [143]. A summary of study associations with an elevation of serum troponin in the context of AECOPD is provided in Table 1.

## 5. Impact of Respiratory Medications and Interventions for AECOPD on Cardiovascular Disease

### 5.1. Bronchodilators

Tachycardia has been shown to be an independent risk factor for cardiovascular mortality in the general population [145]; therefore the liberal use of bronchodilators theoretically could exacerbate underlying cardiac disease during AECOPD. Bronchodilators have beneficial effects by relieving airway disease in the acute setting, but concerns regarding the *β*2 agonist properties and cardiac effects in the setting of a population with high prevalence of cardiovascular disease have resulted in numerous studies to investigate this further.

The Lung Health Study is a large prospective randomized control trial that looked at the benefits of intensified smoking cessation regimens and ipratropium. It found no significant difference in mortality or cardiovascular morbidity associated with ipratropium, although this study was not powered to examine this rigorously [146]. A Cochrane review looked at the use of short-acting *β*2 agonists for stable chronic obstructive pulmonary disease in 2002. They highlight the need for better studies looking at adverse effects secondary to treatment and acknowledge there was not enough information to draw a conclusion [147]. Recently, another Cochrane review was performed comparing short-acting *β*2 agonists to short-acting anticholinergics in patients with stable COPD. The only adverse effects investigated were heart rate and blood pressure, both of which were similar between the two treatment groups once statistical heterogeneity was addressed [148]. Bouvy et al. explored any association between readmission for heart failure exacerbated by an arrhythmia and use of oral and inhaled sympathomimetics. This study was limited by small sample size, so although there was a suggestion of an association of heart failure exacerbation by arrhythmia, the results were not significant [149]. Rossinen et al. prospectively studied 24 patients with known or symptomatic coronary artery disease and comorbid asthma or COPD. After observation for 24 hours on cardiac monitoring while receiving escalating doses of salbutamol, there were no episodes of angina, tachycardia, nor arrhythmias [150].

There are studies that suggest a possible contribution of inhaled bronchodilators to cardiovascular morbidity, but each study has inherent limitations and therefore more studies are needed. The Lung Health Study did not show a statistical difference between supraventricular arrhythmias in patients using ipratropium as compared to those using placebo or with usual care but did find that those with an arrhythmia were very compliant in their usage of ipratropium [146]. A large retrospective case-control study by Wang et al. associates ipratropium use in the preceding thirty days with stroke (OR 2.97; CI 2.27 to 3.88), a risk that diminished if the last prescription was at a longer interval preceding the cerebrovascular event [151]. Dutch citizens with their first nonfatal myocardial infarction were reviewed, finding that patients with ischemic heart disease and a low dose exposure to *β*2 agonists had an increased risk of acute myocardial infarction (OR 2.47, 95% CI 1.60–3.82) [152]. Overall, studies identifying potential adverse effects of bronchodilator use on cardiac disease in the COPD population are small. The evidence that short-acting bronchodilators have adverse cardiac effects is currently lacking.

### 5.2. Noninvasive Positive Pressure Ventilation

Noninvasive positive pressure ventilation (NIPPV), in the form of bilevel positive airway pressure, is a commonly used intervention in severely dyspneic or hypercarbic patients with AECOPD. Recently, 7.5 million admissions for AECOPD between 1998 and 2008 were reviewed documenting an increasing usage of NIPPV from 1% to 4.5% in all admissions for an AECOPD and a significant reduction in the number of patients who required mechanical ventilation [153]. Notably, a subset of COPD patients have negative outcomes associated with NIPPV use, and these were the patients who required mechanical ventilation after failing a trial of NIPPV [153]. Bilevel positive pressure ventilation is ideal for use in AECOPD as it improves arterial oxygenation and arterial hypercarbia at 6, 12, and 24–48 hours after intervention and is associated with a lower likelihood of intubation than those utilizing continuous positive airway pressure (CPAP) [154]. Due to the significant respiratory benefits to utilization of this intervention in AECOPD, it is imperative that we explore the cardiovascular impact of this intervention in a population at such high cardiovascular risk.

Bilevel noninvasive ventilation has been shown to be beneficial in heart failure [155]. NIPPV and continuous positive airway pressure (CPAP) have proven mortality benefits when applied to patients with cardiogenic pulmonary edema as shown in a recent meta-analysis (RR 0.73, 95% CI 0.55–0.97, and RR 0.63, 95% CI 0.44–0.89, resp.) [156]. In both AECOPD and acute heart failure, bilevel noninvasive ventilation increases partial pressure of oxygen in arterial blood, arterial pH within 90 minutes, associated with a reduction in blood pressure [155]. There is a higher frequency of myocardial infarction in those on bilevel who had cardiogenic edema as compared to AECOPD (21% versus 0%) [155]. This finding in itself is not surprising, as ischemia may have been the trigger for acute heart failure, but as to whether bilevel positive pressure ventilation is associated with myocardial infarctions remains to be seen. Mehta et al. studied 27 patients, 13 of whom received nasal CPAP (10 cm H_2_O) and 14 of whom received nasal bilevel positive airway pressure (inspiratory pressure 15 cm H_2_O and expiratory pressure 5 cm H_2_O) [157]. In this cohort, more patients achieved an equivalent if not improved ventilator and hemodynamic response to bilevel positive pressure ventilation, as compared to CPAP, but had a higher frequency of chest pain (10 versus 4) and a higher likelihood of being diagnosed with a myocardial infarction (10 versus 4) [157]. Due to the small cohort size and the difference in symptoms at baseline prior to group assignment, further studies are needed to explore whether this association is valid in the acute setting.

The impact that bilevel ventilation has on hemodynamics has been studied in stable COPD. Sin et al. performed a double-blind, parallel randomized control trial in 23 patients with advanced COPD to determine if bilevel noninvasive ventilation improves cardiac functioning by evaluating heart rate variability, functional performance, and serum markers of cardiac dysfunction after three months of treatment. Notably, use of bilevel positive pressure ventilation resulted in increased heart rate variability, which is a good prognostic marker and a surrogate for improved cardiac functioning as outlined previously [158]. In addition, subjects with daily bilevel positive pressure ventilation had lower NTpro-BNP levels at three months of therapy and a longer 6MWT by 30 m (95% CI 2–57 m) [158]. This study raises important questions regarding the utility of NIPPV in this population, but further studies are needed to confirm and validate these findings with hard clinical outcomes [158]. Notably, similar outcomes were assessed by Held and colleagues when they used right heart catheterization to examine the hemodynamic consequences of NIPPV on patients with pulmonary hypertension secondary to hypoventilation [159]. Five of these 18 patients had COPD as the only cause of hypoventilation [159]. Right heart catheterization at time of initiating bilevel positive pressure ventilation and 3 months after utilizing it daily revealed a decrease in mean PAP, systolic PAP, diastolic PAP, and peripheral vascular resistance with a reduction in right atrial volume and improvement in left atrial volume and right ventricular function with no impact on cardiac index or pulmonary artery wedge pressure [159]. Clinical outcomes monitored revealed substantial improvement in 6MWT (+66 m), maximum work rate (+18 W), and a reduction in NT-proBNP (2487 ± 2143 pg/mL versus 377 ± 416 pg/mL) in comparison to baseline values [159]. Hemodynamic changes associated with low-intensity and high-intensity noninvasive positive pressure ventilation were recently analyzed in a hypercapnic COPD population [160]. A high intensity ventilator strategy averaged 27.6 ± 2.1 cm H_2_O inspiratory positive airway pressure, 4 ± 0 cm H_2_O expiratory positive airway pressure, and respiratory rate of 22 breaths per minute [160]. The high intensity strategy was associated with a reduction in cardiac output, as measured by finometry, which is a possible mechanism that could decrease coronary artery supply in the acute setting [160]. Further work is needed to determine the impact that varying bilevel ventilator protocols have on cardiac physiology and clinically relevant outcomes, so that we optimally manage this high risk population.

## 6. Impact of Cardiac Medications on the Respiratory System during AECOPD

### 6.1. *β* Blockers

The use of *β* blockers in patients with COPD has been a contentious issue since older generations of *β* blockers were shown to be intolerable by patients with obstructive airways disease, by precipitating bronchoconstriction [161], reducing FEV1, or lowering the methylcholine challenge threshold [162]. As a result, *β* blockers have been prescribed less to COPD patients, likely due to a concern that these side effects outweighed any cardiovascular benefit [11, 20, 21, 25]. There is increasing evidence, however, that COPD patients clinically benefit from the use of *β* blockers and may have adverse effects by not taking one. In the presence of comorbid heart disease, COPD patients tolerate heart rates >70 beats per minute poorly reporting more frequent angina, less satisfaction with medical treatment, and a lower quality of life [163].

COPD patients derive significantly less morbidity from respiratory disease when using *β* blockers. A review of 5977 COPD patients in the NHS Tayside Respiratory Disease Information System, Scotland, found that use of *β* blockers was protective for index hospitalization for respiratory disease [164]. For patients taking an inhaled corticosteroid, long acting *β* agonist, and tiotropium in combination with or without a *β* blocker, the hazard ratios were 0.32, 95% CI 0.22–0.44, and 0.70, 95% CI 0.61–0.80, respectively, for respiratory disease admission, and 0.31, 95% CI 0.22–0.43, and 0.68, 95% CI 0.61–0.75, respectively, for prescription of oral corticosteroids [164]. Rutten et al. demonstrated a benefit in prevention of readmission for AECOPD in the subgroup of their cohort without overt cardiovascular disease, as the use of *β* blockers was still protective (HR 0.68, 95% CI 0.46–1.02) [165]. Not all studies have been unanimous in supporting a decrease in respiratory disease admissions. Cochrane and colleagues found an increased annual risk in admission for AECOPD in patients taking *β* blockers that developed over the 6 years of follow-up (risk per annum of acute worsening of symptoms RR 1.30, 95% CI 1.11–1.53 and risk per annum of requiring treatment for an acute exacerbation RR 1.37, 95% CI 1.09–1.72) [166]. Prospective randomized control trials are needed to determine whether there is truly a reduction in hospitalization for respiratory disease while on *β* blocker therapy.

*β* blocker use is associated with a 22% reduction in all-cause mortality (HR 0.78, 95% CI 0.67–0.92) in COPD [164]. A similar association between *β* blockers and an improvement in all-cause mortality following myocardial infarction in the COPD population was found in the VALIANT trial (HR 0.74, 95% CI 0.68–0.80) [21]. A meta-analysis analyzing the mortality benefit of use of *β* blockers in the COPD population identified the most homogenous group as gaining significant benefit from the use of *β* blockers (RR 0.74, 95% CI 0.70–0.79) [167]. The magnitude of reduction is dose dependent, as demonstrated in a Dutch trial of patients undergoing vascular surgery, of whom a third had COPD, where a low dose of cardioselective *β* blocker regimen was associated with a reduced long-term mortality (median follow-up 5 years), and an intensified dose regimen was associated with a reduced 30-day and long-term mortality (low dose being <25% and intensified dose being ≥25% of the maximum recommended dose) [168].

Recent work has focused on determining if there is an optimal *β* blocker of choice in this population. A Cochrane review from 2005 suggested that cardioselective *β* blockers are well-tolerated in COPD patients [169]. A separate meta-analysis looked at studies before May, 2011, and determined that cardioselective *β* blockers only provoked a 30 mL decrease in FEV1 [170]. Lainscak and colleagues performed an open-label randomized control trial of carvedilol and bisoprolol and determined that bisoprolol is associated with an improvement in FEV1 (1561 ± 414 mL versus 1698 ± 519 mL) as compared to carvedilol (1704 ± 484 mL versus 1734 ± 548 mL) and fewer adverse effects (19% with bisoprolol, and 42% with carvedilol) [171]. Discontinuation of the study drug only occurred in two patients for respiratory symptoms [171]. Another study with an open-label, randomized, triple crossover trial between bisoprolol, metoprolol, and carvedilol showed that FEV1 was lowest in the carvedilol group and highest in the bisoprolol group (1.85 L, 95% CI 1.67–2.03 L versus 2.0 L, 95% CI 1.79–2.22 L) [172]. At this time, no *β* blocker has been consistently superior amongst the class 2 and class 3 drugs. Cardioselective *β* blockers are safe to use in COPD patients, initiated at low dose and titrated to effect [173].

### 6.2. Angiotensin-Converting Enzyme Inhibitors (ACEi) and Angiotensin-Receptor Blockers (ARBs)

Several studies have documented a mortality benefit for COPD patients within the first 90 days following discharge from AECOPD who use ACEi and statins [174–176]. Decreased 30-day and 90-day mortality was noted in subjects admitted with AECOPD when taking ACEi (OR 0.58, 95% CI 0.48–0.70, and OR 0.55, 95% CI 0.45–0.66, resp.) [175]. Mancini and colleagues demonstrate a mortality benefit with the use of ARBs after hospitalization for AECOPD and for the combination of ACEi/ARBs and statins [176]. ACEi and ARBs are independently associated with a reduction in myocardial infarction, and both ACEi and ARBs reduced the combined endpoint of MI or death [176]. Further evidence is needed as to whether this mortality benefit is purely due to a decrease in cardiovascular mortality.

There has been an effort to determine whether ACE inhibitors may directly improve morbidity from COPD. The DD genotype of the angiotensin-converting enzyme (ACE) is associated with pulmonary hypertension and tissue oxygenation with exercise, raising questions as to whether it is associated with the phenotype of COPD [177]. Captopril administered to COPD patients resulted in improved mean PAP, peripheral vascular resistance, and lactate production in genotypes other than the DD genotype, as measured by right heart catheterization, suggesting captopril positively influenced exercise performance [178]. Recently, Zhang and colleagues looked at ACE gene polymorphisms and exercise performance in patients with COPD and found no significant differences in resting lung function and cardiopulmonary exercise testing parameters among the three ACE genotype control groups [179]. Di Marco et al., in 2010, performed a double-blind, placebo-controlled, crossover study looking at 21 patients with moderate to severe COPD by GOLD criteria using enalapril 10 mg daily or placebo for four weeks. The authors showed a significant improvement in work rate at peak and anaerobic threshold and increased cardiac stroke volume. The only spirometric value that was significantly improved was DLCO adjusted for alveolar volume [180]. For clinical outcomes such as reduction in admission for AECOPD, ACEi and ARBs were only correlated with this when combined with statin use [176]. At present time, further studies are needed to define the role that ACEi and ARBs can perform to reduce the morbidity and mortality of respiratory and cardiovascular comorbidity in the COPD population.

### 6.3. Statins

Originally designed to lower cholesterol, the 3-hydroxy-3-methylglutaryl coenzyme A (HMG-CoA) reductase inhibitors, also called “statins,” are recognized as anti-inflammatory agents [181] and have been demonstrated to decrease morbidity from COPD. Experimental observations suggest that these agents have a wide range of pleiotropic anti-inflammatory properties *in vitro *and* in vivo* with the inhibition of isoprenoid synthesis, which leads to the inhibition of small GTPases such as Rho, Rac, and Cdc42, making them potentially useful in COPD [182–185]. Lee et al. in 2008 performed a prospective, double-blind, randomized control trial on 125 stable COPD patients comparing placebo and pravastatin 40 mg daily and demonstrated a significant improvement in lipid levels and hsCRP. Exercise capacity was significantly improved in those taking pravastatin, from 599 ± 323 s to 922 ± 328 s, as compared to stable exercise times in the placebo group (608 ± 273 s to 609 ± 180 s) with no improvement in lung function parameters [186]. These findings suggest that curbing lung and systemic inflammation with the use of a statin may improve quality of life, exercise capacity, and chronic inflammation in stable COPD [186]. Bartziokas and colleagues also looked at morbidity from COPD and demonstrate an association between statin therapy and an improvement in health-related quality of life and fewer AECOPD (2.1 ± 2.7 versus 2.8 ± 3.2 AECOPD/patient, resp.) [187]. This is supported by Mancini and colleagues work that reports a decreased risk of hospitalization for AECOPD when statins are combined with either an ACEi or ARB [176]. A prospective randomized control trial is currently underway with the aim to support these findings.

Statins are associated with improved mortality from COPD. A systematic review performed by Dobler and colleagues supports the mortality benefit of statins in COPD, but the data was predominantly based on observational and retrospective studies, with only the one randomized control trial by Lee et al. [186, 188]. Since this review, Bartziokas et al. have published another prospective study that showed no difference in mortality at 30-days and one year [187]. Two studies retrospectively looked at mortality and statin use, data not incorporated into the review, and found lower mortality from COPD in those on statin therapy [175, 189]. At this time, there is some evidence to support a mortality benefit in COPD with statin use, but further prospective studies are needed.

## 7. Potential Mechanisms of How AECOPD Could Trigger Cardiovascular Disease/Events

**Figure 2:**
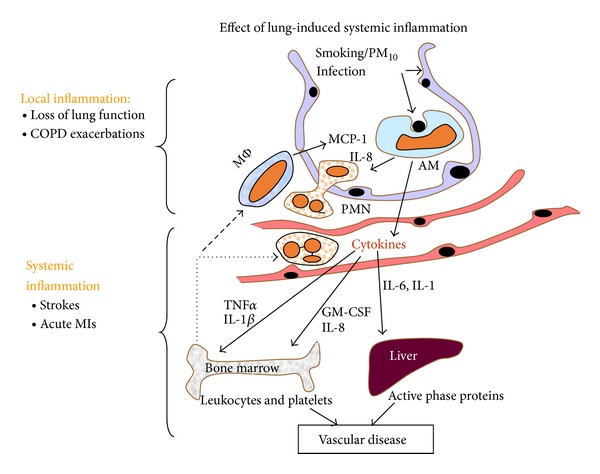
Acute inflammatory events in the lungs provoke a cascade of systemic inflammation that starts in the lung, with hematologic spread to other organs, activating the systemic inflammatory response, and thereby promoting the development of atherosclerosis and vascular events.

There are strong mechanistic links between acute and chronic lung injury, inflammation, peripheral vascular disease, acute vascular events [97–99], and endothelial dysfunction [51]. COPD is characterized by chronic inflammation in lung tissue and the extent of the inflammatory reaction correlates with the severity of the disease [190]. Chronic inflammation in the lung parenchyma is associated with a downstream systemic inflammatory response [191] characterized by activation of the acute phase response, release of inflammatory mediators in the circulation, stimulation of the bone marrow to release leukocytes and platelets, and priming and activation of circulating leukocytes and vascular endothelium. While this systemic inflammatory response impacts many organ systems, the vascular system is particularly affected.  Figure 2 shows how lung inflammation triggers an inflammatory cascade, causing potential downstream effects of this systemic inflammatory response on blood vessels while enhancing the inflammatory response in the lung, thereby initiating a vicious cycle.

**Figure 3:**
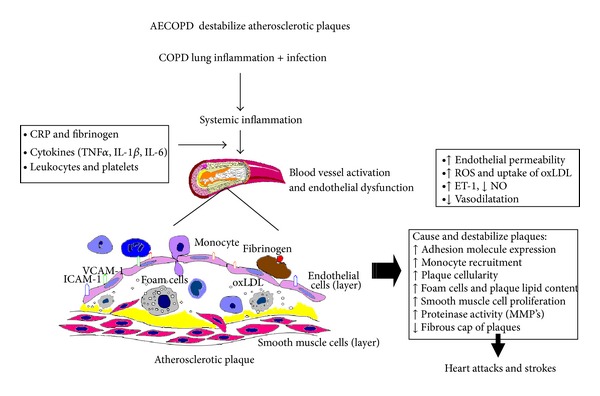
AECOPD is associated with an acute lung injury initiating the local and systemic inflammatory pathways that cause endothelial injury and vascular dysfunction, a prothrombotic environment, and instability in vascular plaques that may predispose to coronary and cerebrovascular events.

Recent experimental evidence has shown that inflammatory mediators produced in the lung (following exposure to either lipopolysaccharide or particulate matter) directly translocate to the blood stream supporting the concept that inflammation in the lung directly contribute to the downstream systemic response [192, 193]. The leakage of inflammatory mediators, such as reactive oxygen species, cytokines, and chemokines generated in the lung airspaces and lung tissues, directly into the peripheral blood, could activate all of the different pathways characteristic of a systemic inflammatory response. Importantly, this includes peripheral blood leukocytes and blood vessel activation resulting in progression of atherosclerosis with downstream cardiovascular events, the predominant reason for COPD morbidity and mortality. Several studies have shown that this systemic inflammatory response in COPD is augmented during an acute exacerbation, leaving the vasculature and atherosclerotic plaques even more vulnerable for activation, rupture, and thrombus formation, resulting in acute cardiac events [191]. Lastly, recent experimental evidence showed that lung inflammation [194] and vascular activation and atherosclerosis induced by airborne particles can be attenuated by statins [195, 196], supporting clinical data that statins improve COPD symptoms and reduce AECOPD.  Figure 3 shows potential mechanisms of how acute exacerbation of COPD could trigger systemic inflammation that activate the vasculature and specifically atherosclerotic plaques, inducing endothelial dysfunction, plaque instability, and a prothrombotic state that together could trigger vascular events.

## 8. Conclusion

COPD is a complex lung and systemic disease that is associated with a variety of cardiovascular diseases including coronary artery disease, peripheral vascular and cerebrovascular disease. COPD patients have frequent right and left ventricular dysfunction and an increase in sympathetic activation with high morbidity from arrhythmias. Acute exacerbations of COPD may trigger cardiac events but are also often precipitated by cardiac events. At the present time, many of these events are unrecognized, despite improved tools for diagnosis and assessment. The treatments we utilize for AECOPD have not been rigorously examined as to the effects they have on a vulnerable cardiovascular system and further studies are needed to explore this area. Finally, the association between lung inflammation (including that in COPD and AECOPD) and cardiac events may be due to consequences of the systemic inflammatory response and downstream microvascular changes in plaque stability, hypercoagulability, and endothelial cell dysfunction.

## Figures and Tables

**Table 1:** Summary of studies analyzing the prognostic and diagnostic importance of serum troponins in patients admitted to hospital for acute exacerbations of COPD.

Author	Study design	Sample size	Controls	Follow-up (months)	Associations with elevated troponin
Fruchter and Yigla [83] Israel	Retrospective	83	953 without troponins and 99 with low cTnI	≤72	Increased long-term mortality

Martins et al. [95] Portugal	Retrospective	121	52 with undetectable cTnI	>18	Age, heart failure, atrial arrhythmia, elevated BNP, need for NIPPV Increased LOS and long-term mortality

Harvey and Hancox [96] New Zealand	Retrospective	47	147 with undetectable cTnI or cTnT	Until discharge	Older age, lower pulse oximetry, acidosis, hypercapnea Longer length of stay in hospital

Brekke et al. [138] Norway	Retrospective	173	897 without troponins and 223 with undetectable cTnT	<66	Increased all-cause mortality (HR 1.64, 95% CI 1.15–2.34)

Brekke et al. [143] Norway	Retrospective	321	120 with undetectable cTnT	Discharge	Neutrophilia, increased creatinine, cardiac infarction injury score, low hemoglobin, and tachycardia

Odigie-Okon et al. [137] USA	Prospective	19	95 without an ischemic ECG and undetectable cTnT	First 24 hours from admission	Presence of acute coronary syndrome or marker of ischemia

Marcun et al. [87] Slovenia	Prospective	32 at admission 15 at discharge	95 admitted with cTnT ≤ 0.012 ng/L and 112 discharged with cTnT ≤ 0.012 ng/L	6	Increased risk of repeat hospitalization (HR 2.89, 95% CI 1.13–7.36)

Høiseth et al. [139] Norway	Prospective	73	26 with a low highly sensitive cTnT	<36	Increased long-term mortality and higher mortality with tachycardia

Høiseth et al. [141] Norway	Prospective	49	50 with a low geometric mean of hs-cTnT	Discharge	Age, arterial hypertension, tachycardia, creatinine

Baillard et al. [142] France	Prospective	13	58 with normal troponins	Discharge	In-hospital mortality

Soyseth et al. [136] Norway	Prospective	50	124 stable COPD patients admitted to a rehabilitation hospital	Until discharge	No association between retrosternal chest pain or T wave inversions on ECG and elevated troponin

Chang et al. [144] New Zealand	Prospective	40	201 with undetectable cTnT	12	Increased 30-day mortality

LOS: length of stay; LVH: left ventricular hypertrophy; cTnI: cardiac troponin I; cTnT: cardiac troponin T; AECOPD: acute exacerbation of COPD; ECG: electrocardiogram.
